# Enteric Pathogens and Their Toxin-Induced Disruption of the Intestinal Barrier through Alteration of Tight Junctions in Chickens

**DOI:** 10.3390/toxins9020060

**Published:** 2017-02-10

**Authors:** Wageha A. Awad, Claudia Hess, Michael Hess

**Affiliations:** 1 Clinic for Poultry and Fish Medicine, Department for Farm Animals and Veterinary Public Health, University of Veterinary Medicine Vienna, 1210 Vienna, Austria; claudia.hess@vetmeduni.ac.at (C.H.); michael.hess@vetmeduni.ac.at (M.H.); 2 Department of Animal Hygiene, Poultry and Environment, Faculty of Veterinary Medicine, South Valley University, Qena 83523, Egypt

**Keywords:** paracellular permeability, tight junction, intestinal barrier, leaky gut, enteric pathogens, gut health, chickens

## Abstract

Maintaining a healthy gut environment is a prerequisite for sustainable animal production. The gut plays a key role in the digestion and absorption of nutrients and constitutes an initial organ exposed to external factors influencing bird’s health. The intestinal epithelial barrier serves as the first line of defense between the host and the luminal environment. It consists of a continuous monolayer of intestinal epithelial cells connected by intercellular junctional complexes which shrink the space between adjacent cells. Consequently, free passing of solutes and water via the paracellular pathway is prevented. Tight junctions (TJs) are multi-protein complexes which are crucial for the integrity and function of the epithelial barrier as they not only link cells but also form channels allowing permeation between cells, resulting in epithelial surfaces of different tightness. Tight junction’s molecular composition, ultrastructure, and function are regulated differently with regard to physiological and pathological stimuli. Both in vivo and in vitro studies suggest that reduced tight junction integrity greatly results in a condition commonly known as “leaky gut”. A loss of barrier integrity allows the translocation of luminal antigens (microbes, toxins) via the mucosa to access the whole body which are normally excluded and subsequently destroys the gut mucosal homeostasis, coinciding with an increased susceptibility to systemic infection, chronic inflammation and malabsorption. There is considerable evidence that the intestinal barrier dysfunction is an important factor contributing to the pathogenicity of some enteric bacteria. It has been shown that some enteric pathogens can induce permeability defects in gut epithelia by altering tight junction proteins, mediated by their toxins. Resolving the strategies that microorganisms use to hijack the functions of tight junctions is important for our understanding of microbial pathogenesis, because some pathogens can utilize tight junction proteins as receptors for attachment and subsequent internalization, while others modify or destroy the tight junction proteins by different pathways and thereby provide a gateway to the underlying tissue. This review aims to deliver an overview of the tight junction structures and function, and its role in enteric bacterial pathogenesis with a special focus on chickens. A main conclusion will be that the molecular mechanisms used by enteric pathogens to disrupt epithelial barrier function in chickens needs a much better understanding, explicitly highlighted for *Campylobacter jejuni, Salmonella enterica* and *Clostridium perfringens*. This is a requirement in order to assist in discovering new strategies to avoid damages of the intestinal barrier or to minimize consequences from infections.

## 1. Introduction

Epithelial cells are tightly bound together by intercellular junctional complexes that regulate the passage of ions and molecules through the paracellular pathway. Reduced tight junction integrity greatly increases ion conductance across the paracellular route compared to the transcellular route, resulting in a phenomenon described as leaky gut [1]. This condition basically enables pathogens and endotoxins to access the whole body including vital organs.

Di Pierro [2] reported that the opening and closing of the paracellular junction is tightly regulated, under normal conditions. However, dysregulation and loss of the cellular junction integrity contributes to disease development. The degree of sealing of tight junctions varies according to external stimuli, physiological and pathological conditions.

Tight junctions are multi-protein complexes that not only hold cells of a same tissue together but also form channels which allow permeation between the cells, resulting in epithelial surfaces of different tightness. The main component of tight junctions proteins are occludin, tricellulin, and claudins. Tight junctions are regulated in their molecular composition, ultrastructure, and function by intracellular proteins and the cytoskeleton. Consequently, TJs play a crucial role in the physiological function of epithelial cells.

In general, changes in gut permeability can be induced via modulation of TJs (down or up-regulation of the TJ proteins), or relocation of TJs or/and cytokine and hydrogen peroxide-induced decrease in transepithelial tissue resistance [3]. Hecht [4] showed that enteric pathogens target the intercellular tight junctions and can disrupt them either directly by affecting specific TJ proteins or indirectly by altering the cellular cytoskeleton (through changes in the perijunctional actomyosin ring). Disruption of specific TJ proteins can result from degradation by bacterial derived proteases or by biochemical alterations such as phosphorylation or dephosphorylation.

The barrier function of TJs and intestinal permeability can be directly determined in vitro with mounted tissue in the Ussing chamber technique based upon a decrease in transepithelial electrical resistance (TEER) and an increase in the paracellular flux of macromolecules such as mannitol, reflecting a quantifiable indicator for the intestinal barrier [5,6]. The barrier function in vivo may also be assessed indirectly by characterizing TJ proteins or by serological detection of substances such as bacterial lipopolysaccharides (LPS) in the blood [7] (Table 1).

The assessment of tight-junction integrity is complex, which is reflected by the finding that not only the quantity of mRNA but also phosphorylation and folding together with localization of TJ proteins are of importance [11,12]. However, most of these features are poorly understood and deserve more detailed investigations.

In general, pathogens can disrupt the tight junctions’ barrier function by different mechanisms including direct reorganization or degradation of specific TJ proteins, reorganization of the cell cytoskeleton, and activation of host cell signaling events [13]. Additionally, it was reported that some enteric pathogens appear to influence tight junction functions by utilizing TJ proteins as receptors for internalization and breakdown of the epithelial barrier [14]. Consequently, it can be summarized that enteric pathogens can develop a broad range of mechanisms to change the host tight junction barrier function. Furthermore, it was reported that pathogen induced alterations of the actin cytoskeleton through modification of host cell pathways, such as the activation of myosin light chain kinase (MLCK), contraction of the perijunctional actomyosin via phosphorylation of MLC by MLCK, alters the activity of the Rho family of GTPase binding proteins, which are involved in the assembly and/or organization of the actin cytoskeleton [15,16,17].

Finally, TJ proteins have a dominant role in barrier formation, as resolved mainly from work with mammals. Based upon nutrient uptake, permeability studies and the way chickens react to microorganisms it appears that the constitution of the epithelial barrier in birds is somewhat different to that in mammals [18,19,20,21,22], which might help to explain differences in the clinical outcome following infection with the same kind of pathogen. Consequently, resolving the structure of TJs in the chicken gut would help to elucidate how compartmental separation and transepithelial transport takes place at different age of the animals, keeping in mind that the constitution of tight junctions can be used as a marker for gut health and integrity. A better knowledge of the composition of TJ proteins in chickens is also crucial to understand certain pathogenic pathways.

The following sections will not only outline the molecular structure and function of tight junctions, disruptions of TJ proteins by enteric food borne pathogens will also be addressed due to their importance for birds’ health. In addition, an overview will be provided about the strategies used for restoration of the impaired barrier permeability.

## 2. Molecular Structure and Function of Tight Junctions

**Figure 1:**
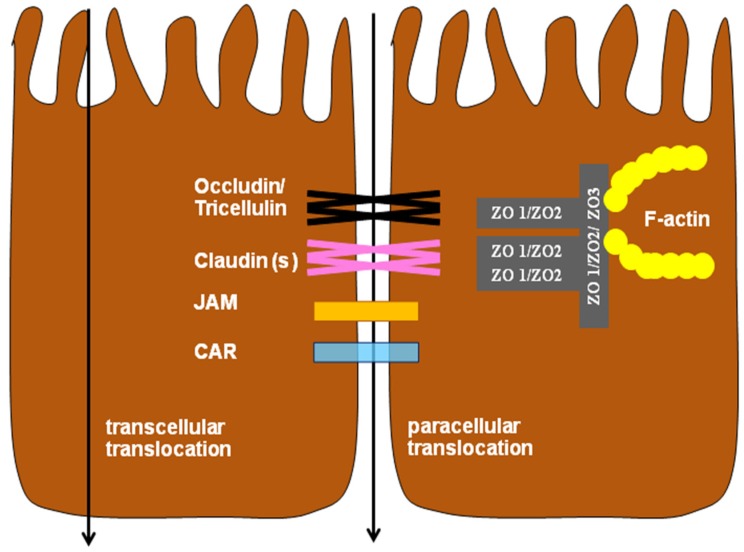
Schematic outline of the principle pathways (transcellular and paracellular) of translocation across the intestinal epithelium with tight junction proteins. JAM = Junctional adhesion molecule, CAR = Coxsackie virus and adenovirus receptor, ZO = Zonula occludens (adapted from Ulluwishewa et al. [24]).

Generally, TJs are multi protein complexes consisting of transmembrane proteins, linked to the actin cytoskeleton via cytoplasmic proteins [23]. Approximately 50 TJ proteins have been identified. Transmembrane proteins, principally claudins, occludin, junctional adhesion molecules (JAMs), the coxsackie virus and adenovirus receptor (CAR) and tricellulin (Figure 1), are contributing to the semi-permeable barrier, whereas cytosolic proteins not only link membrane components to the actin cytoskeleton they also participate in signaling between TJs and the cell nucleus.

Proteins of the claudin family are a main component of tight junctions and form a seal that modulates paracellular transport in the intestinal epithelium [25]. It was also suggested that claudins have an important role in the regulation of cellular signaling [26,27]. Claudin-1, -3, and -5, and cldn-16, ZO-1 and ZO-2 expressions were demonstrated in the chicken intestinal epithelium [26,28,29,30]. It is known that claudin-1, -3, -4, -5, -7, and -19 are pore-sealing claudins. An increased expression of these proteins leads to a very tight epithelia, coinciding with an increased transepithelial electrical resistance (TER) and decreased solute permeability (mainly sodium ions) across the epithelial monolayer [31,32,33]. Conversely, claudin-2 and -15 are considered as the pore-forming claudins, because of their ability to form paracellular anion/cation pores as well as water channels, enabling them to decrease epithelial tightness and to increase solute permeability by allowing the passage of sodium ions [34,35,36]. Taken together, claudins enable strict control over the paracellular flux of cations and anions [37].

Occludin is a TJ protein consisting of four transmembrane domains with the capability to shift to various paracellular locations and, therefore, altering epithelial permeability. Movement of occludin from the tight junction into cytoplasmic vesicles occurs frequently during barrier function loss [38] and has been shown to be triggered by multiple stimuli, such as oxidative stress and inflammation [39]. Cani et al. [11] showed that occludin expression is inversely correlated with the translocation of Fluorescein isothiocyanate (FITC) dextran from the gastrointestinal tract to the blood, emphasizing its importance in maintaining the barrier function.

Zona occludens-1 (ZO-1) was the first protein identified at tight junctions. It localizes at the cytoplasmic surface of the cell membrane, close to the TJ strands [40,41]. There are three ZO types: ZO-1, ZO-2 and ZO-3. ZO-1 plays a major role in the formation of TJs in epithelial cells compared with ZO-2 and ZO-3 [35]. Furthermore, ZO-1 serves as an important linker between the TJ and the actin cytoskeleton and is thought to be a functionally critical tight junction component. It was also found that ZO-1 is directly associated with occludin [42].

A fourth transmembrane protein, tricellulin, has also been recently discovered, a tight-junction protein forming a linkage between three adjacent cells [43]. Tricellulin is found concentrated at tricellular contacts in epithelial cellular sheets identified in epithelial cells of the kidney, intestine and stomach [43,44]. Tricellulin is a tetra-span protein with four transmembrane domains and two extracellular loops. Currently the role of tricellulin at TJs is largely unknown.

Paracellular permeability across the intestinal epithelium is regulated by tight junctions [32,45]. Over the past few years, many studies focused on identifying the mechanisms that permit a selective diffusion of ions and solutes along the paracellular pathway and much knowledge about the molecular composition of TJs has been provided [23]. Each TJ protein has a specific function which has just started to be elucidated [46]. For example, JAMs were shown to play a role in tight-junction formation, but not in barrier maintenance [47].

Tight junctions promote two functions (fence and screening functions) which are crucial for an appropriate epithelial function. The fence function is vital in maintaining apical and basolateral character, whereas the screening function acts as a gatekeeper, regulating paracellular transport of solutes between the luminal and basolateral space [48].

Finally, tight junctions are regulated by several intracellular pathways including myosin light chain kinase (MLCK), mitogen-activated protein kinases (MAPK), protein kinase C (PKC) and the Rho family of small GTPases [24]. The MLCK pathway is one of the most abundant in the gut, and is a crucial step in the regulation of tight-junctional permeability by several external stimuli, such as cytokines and pathogens [15] and the inhibition of MLCK prevents the deterioration of barrier function. To understand how tight junction proteins change in the course of barrier dysfunction, it is important to analyze such multiple proteins, in order to understand their interactions and to determine the activation status of regulatory pathways.

## 3. Infection and Inflammation Disrupt Barrier Function

The main entry site for pathogenic bacteria, feed contaminants such as mycotoxin and other pathogens is the digestive tract. On the contrary, the intestine forms a major physical barrier preventing pathogens and toxic compounds to cross the mucosa and to enter the body, coinciding with the activation of the immune system (innate and adaptive immune responses) against pathogens and toxic compounds. Thus, intestinal integrity is critical for maintaining a physical barrier between the intestinal lumen and the body and to protect against infection. The barrier function of TJ is of critical importance in gut physiology. TJs also play important roles in signal transduction mechanisms that regulate cell proliferation, differentiation and gene expression [49]. Under normal physiological conditions, tight junction barrier integrity remains intact and transport of toxic luminal substances together with molecules across the tight junctions is very well regulated [50].

Shen et al. [51] reported that different factors can affect the permeability of the intestinal tight junction barrier. They demonstrated that small quantities of luminal endotoxin, commensal microflora and pathogens may cross the epithelium and enter circulation through the tight junctions, when animals are under stress or suffer from an intestinal inflammation. Pathogens can also stimulate the localized secretion of pro-inflammatory cytokines from immune and intestinal epithelial cells. Consequently, these inflammatory and stress responses may induce phosphorylation of myosin light chain by myosin light chain kinase, resulting in contraction and opening of the intestinal epithelial tight junctions and an increased intestinal permeability [52,53]. In this context, it needs to be mentioned that heat stress is of high importance in poultry production and an influence on broilers physiology was demonstrated, as stressed birds suffer from multiple physiological disturbances such as damages to intestinal mucosa and higher intestinal paracellular permeability [54].

Some bacterial pathogens can impair intestinal barrier function by disruption of tight junctions and initiation of inflammatory cascades [55]. In addition, most of them attack epithelial cells either directly using effector proteins or through the elaboration of enterotoxins. Berkes et al. [56] reported that many enteropathogenic bacteria have been implicated in the disruption of tight junctions including enteropathogenic *Escherichia coli* (EPEC), *Clostridium difficile*, *Clostridium perfringens*, *Helicobacter pylori*, *Campylobacter jejuni*, *Campylobacter concisus*, and *Salmonella* Typhimurium. Some of these bacteria disrupt tight junctions through disorganization of specific tight junction proteins, including zonula occludens, occludin, and claudin [57]. It was also demonstrated that some of them, such as pathogenic *E. coli*, cause a withdrawal of ZO-1, occludin and claudins from the TJ. Furthermore, many of the barrier-disruptive mechanisms were reported such as dephosphorylation of occludin [58], decreased junctional protein expression [59] and stimulation of non-muscle myosin through myosin light chain kinase (MLCK) [60] and Rho GTPases [61].

There is evidence demonstrating a role of pro-inflammatory cytokines, such as interferon-γ (IFN-γ) and tumour necrosis factor-α (TNF-α), in endocytosis of tight junction proteins from the apical junctional complex (AJC) through MLCK and Rho-associated kinase (ROCK)-mediated manipulation of the host cell cytoskeleton [62,63,64]. In addition, pro-inflammatory stimuli trigger intestinal epithelia to express more of the relatively permeable tight junction proteins (e.g., claudin-2) and less of the relatively impermeable junction proteins (claudins-1, -3, -4, -5, and -8), resulting in a decreased barrier function [65,66]. Epithelial barrier dysfunctions occur in inflammatory bowel diseases that contribute to leaky-flux diarrhea, coinciding with a loss of solutes and water. Furthermore, down-regulation of pore-sealing claudins (4, 5, and 8), but up-regulation of pore-forming claudin-2 is observed in Crohn’s disease [66,67].

Moreover, it was shown that bacterial toxins, such as endotoxins of Gram-negative bacteria (LPS), could induce disorders in intestinal epithelial barrier function [7]. Therefore, intestinal and systemic diseases are associated with leaky epithelial barrier and consequently increased intestinal permeability to endotoxin. Additionally, Albin et al. [68] showed that endotoxins can alter the intestinal integrity and junctional organization. Finally, the disruption of gut barrier could induce a malabsorption of nutrients and translocation of enteric bacteria to various internal organs, leading to disease and reduced growth performance [5,69,70,71,72].

Generally, it can be concluded that an impaired gut barrier function is a common characteristic of many local but also systemic infections and a leaking gut is thought to contribute to the severity of clinical symptoms. Finally, enteric pathogens utilize a diverse array of strategies to alter host tight junction barrier function and such alterations can contribute to different infection outcomes. Thus, mechanisms whereby certain enteric pathogens disrupt the tight junctional complexes will be addressed below in more detail and how, in turn, these disruptions may be implicated in gastrointestinal dysfunction.

### 3.1. Enteropathogenic Escherichia coli (EPEC)

Enteropathogenic *E. coli* are a major cause of bacterial diarrhea and hemorrhagic colitis in both humans and animals [73,74,75] with consequences on intestinal epithelial barrier function. Ugalde-Silva et al. [76] reported that EPEC injects effector proteins directly from the bacterial cytoplasm to the host cell cytoplasm and thereby alters the eukaryotic cell functions through modifying or blocking cell signaling pathways. Philpott et al. [77] demonstrated in an in vitro study that EPEC induces a time and dose dependent drop in tissue resistance across intestinal epithelial cell monolayers with an increase in the paracellular permeability.

Furthermore, Muza-Moons et al. [78] showed that the tight junction proteins occludin, claudin and ZO-1 are affected by an EPEC infection in T84 intestinal epithelial cells. Roxas et al. [79] reported that *E. coli* induced changes in intestinal ion permeability in the colon of mice were due to alterations in tight junction architecture. Applying immunofluorescence microscopy, a redistribution of the tight junction proteins occludin and claudin-3 together with an increased expression of claudin-2 could be demonstrated. Furthermore, it was demonstrated that the acute exposure to *E. coli* (enteric non-pathogenic and pathogenic) reduced the epithelial ion conductance [80]. It was also found that an infection with EPEC reduced significantly the TJ proteins (occludin (phosphorylated form), ZO-1 and claudin-1), which is supported by other findings reporting a rapid and progressive dephosphorylation of occludin following EPEC infection [58].

Hofman [81] showed that bacterial toxins have the ability to dilate TJs and increase paracellular permeability. These effects may result from direct modification of TJ proteins (occludin, claudins, ZO-1, ZO-2, ZO-3) or by direct binding to a TJ component or by alteration of the peri-junctional actin filaments, keeping in mind that overlapping effects may appear. It was reported that the EPEC secreted effector protein F (EspF) is necessary for disrupting the tight junction barrier function in vitro [82] (Table 2). A study using epithelial cell lines has demonstrated that an EPEC infection leads to a decrease in TER as well as a disruption of tight junction barrier function through redistribution, dephosphorylation and dissociation of tight junction proteins [82,83]. In an in vivo study, it was mentioned that the EPEC-induced tight junction barrier disruption is EspF dependent at earlier time points of infection, while altered barrier function at the later time point was shown to coincide with increased production of the pro-inflammatory cytokine TNF-alpha [84,85]. Altogether, it can be summarized that the intestinal epithelial response to infection can be multifactorial.

### 3.2. Campylobacter jejuni

Different studies demonstrated that *C. jejuni* can disrupt the structure of tight junction proteins, in order to facilitate their paracellular passage into the underlying tissues [86,87,88]. The mechanisms by which *C. jejuni* affects tight junction functions are summarized in Table 3. In one of these studies, it was demonstrated that *Campylobacter* can affect the intestinal integrity by disrupting occludin, an integral tight junction protein, enhancing the paracellular passage of *Campylobacter* in Caco-2 cell monolayers [86]. Accordingly, the host will elevate levels of pro-inflammatory cytokines, such as TNF-α and IFN-γ which have been shown to affect the structure of tight junctions, to disrupt the barrier function and to facilitate the passage of luminal antigens into the underlying tissues [87]. In addition, paracellular leakage contributes to a disturbance of selective intestinal transport (e.g., toxin absorption) and diarrhea [89].

In another in vitro study with Caco-2 cells [90] it was demonstrated that *C. jejuni* is capable to enter host eukaryotic cells via endocytosis. In the same study it was also revealed that *C. jejuni* 81116, in the presence of IFN-γ alone or with TNF-α, resulted in a focal redistribution of occludin and increased cellular damage within 24 h. Furthermore, co-infection of *C. jejuni* and *E. coli* caused a significant decrease in TEER within 6 g, with a focal redistribution of occludin, correlating with an influx of *C. jejuni* into the basal side of enterocytes. Dodson [90] also hypothesized that once *Campylobacter* colonized the gastrointestinal tract of a susceptible host, the infection causes an intestinal inflammation which would result in a rapid loss of tight junction barrier function. Similarly, other studies showed that *C. jejuni*-induced barrier dysfunction was associated with altered claudin-4 expression and distribution [91,92].

Recently, some studies showed that *C. jejuni* promotes the translocation of *C. jejuni* itself as well as other commensal bacteria in mammals and chickens [70,93,94]. It is supposed that the intestinal bacteria target various intracellular pathways, change the expression and distribution of TJ proteins and thereby alter gut permeability. In addition, this pathogen can affect the gut barrier functions by inducing fluid and electrolyte secretion and initiate inflammatory responses [56]. Hence, it was shown that *C. jejuni* infections in some broiler lines (fast-growing lines) are characterized by diarrhea, a prolonged inflammatory response and induction of lymphocyte activation in cecal tissue [95]. Moreover, *C. jejuni* colonization was associated with an alteration of the gut microbiota with changes in bacterial metabolic activity (short-chain fatty acids, SCFAs) [70,71]. In a recent study, we showed that *Campylobacter* infection strongly interferes with Ca^2+^ signaling [69]. It can be hypothesized that such an interaction of *C. jejuni* with [Ca^2+^] can have profound effects on cellular functions and may support cellular invasion of *Campylobacter* by microvillar cytoskeleton rearrangement which needs further approval.

**Figure 2:**
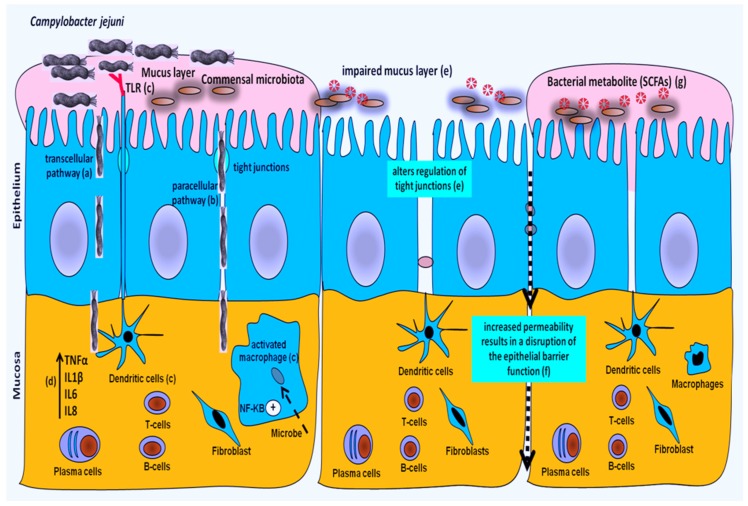
Pathophysiology of *Campylobacter* in chickens: translocation via transcellular (a) and paracellular pathways (b). Macrophages and dendritic cells (innate immune cells) recognize the pathogenic bacteria through molecular pattern-recognition receptors (Toll-like receptor, TLR) (c), change their functional status from tolerogenic to an activated phenotype. Activation of nuclear factor-κB (NF-kB) pathway stimulates gene transcription, resulting in increased production of pro-inflammatory cytokines (TNF-α, interleukins 1β, IL 6 and IL8) [95] (d). *Campylobacter* induces a disruption of tight junctions and the mucus film (e) with a higher permeability of the intestinal epithelium (f), resulting in an increased uptake of luminal antigens (e.g., microbes, and toxins). In addition, *Campylobacter* utilizes SCFAs as a source of carbon and energy in the intestine, consequently alters gut colonization dynamics and may also influence physiological processes due to altered microbial metabolite profiles [70] (g).

Chickens are recognized as an imperative source of thermophilic *Campylobacter*, carrying this pathogen in their intestinal tract. Recently, it was revealed that *C. jejuni* colonization in the chicken intestine was accompanied with mucosal damage and a higher intestinal permeability which indicates that *C. jejuni* may translocate via the paracellular, in addition to the transcellular, pathway [5,69,95,96] (Figure 2). Although both pathways can be involved in bacteria translocation, the paracellular pathway appears to be of particular importance in dissemination towards inner organs. In chickens, it can be hypothesized that the barrier function of the intestinal epithelium is markedly altered in colonized birds and this alteration could be a part of the colonization strategy leading to persistent infection of the gut [97]. However, the mechanisms involved in *C. jejuni*-induced barrier dysfunction in chickens remain unclear and further studies are needed to determine how *Campylobacter* influences the gut barrier during infection. Additionally, elucidating the mechanisms by which *C. jejuni* is able to cross the gut should help in finding suitable options to decrease the level of bacteria in internal organs.

### 3.3. *Salmonella enterica*

*Salmonella* is another important food-borne pathogen with limited clinical signs in chickens, although intestinal inflammation and elevated cross-contamination are noticed [98,99,100]. Infection of epithelial cell monolayers by *S.* Typhimurium resulted in a disrupted TJ structure and function [16,17,101,102,103] (Table 4). Similarly, in an in vitro experiment it was demonstrated that the infection of T84 intestinal epithelial cells with *S.* Typhimurium elicited a rapid drop in tissue resistance, accompanied by an increase in the paracellular flux of fluorescence labeled markers across the infected cell monolayer [103]. Coinciding with the increased paracellular permeability, *Salmonella* caused a decrease in the expression of both ZO-1 and phosphorylated occludin, redistribution of claudin-1 and ZO-2 proteins, facilitation of bacterial translocation, and loss of barrier function [103].

It has also been described that *S.* Typhimurium invasion of intestinal epithelia is accompanied by a loss of epithelial integrity and, consequently, an impaired epithelial function in mice [57,104]. Moreover, an infection of intestinal epithelial cells (T84 or MDCK) with *S.* Typhimurium can cause a progressive decrease in transepithelial electrical resistance, alteration of intestinal TJ proteins, a damage of intestinal barrier function and facilitates the translocation of both pathogenic and non-pathogenic bacteria across epithelial cell monolayers, indicating a disruption of the tight junction barrier [102,103]. It was further reported that the increased paracellular permeability following a *S.* Typhimurium infection can be due to the contraction of the perijunctional actin ring and alteration in the Rho GTPase activity via the type three secretion system (T3SS) effector proteins, thereby altering the function of tight junctions [16,17,102].

In a similar way, Zhang et al. [100] found that a *S.* Typhimurium challenge decreased claudin-1 and occludin mRNA expression in the ileum of broiler chickens. Moreover, it was reported that the intestinal tight junction proteins claudin-1, claudin-4 and occludin mRNA expression in the jejunum at 14 days post infection (dpi) were significantly decreased by a *S.* Typhimurium challenge in broilers [105]. This down-regulation of TJ proteins resulted in an enhancement of paracellular permeability and disruption of the intestinal barrier, thereby allowing the diffusion of macromolecules, such as bacterial toxins (endotoxin) and pathogens, from the intestinal lumen into the blood circulation [106,107].

Like *S.* Typhimurium, *Salmonella* Enteritidis could also alter the tight junction function. Awad et al. [20] found that luminal *S.* Enteritidis affects the intestinal epithelium of chickens in the same way as its endotoxin and it decreases intestinal ion permeability of chickens directly after acute exposure. This is in contrast to findings in pigs where *Salmonella* endotoxin does not elicit an acute decrease in permeability [108]. This finding could explain why chickens do not experience overt secretory diarrhea when infected by this pathogen in contrast to pigs and other species, including humans, which is seen as a result of a differently regulated gut function. Finally, intestinal TJ disruption results not only in an increased permeability to luminal antigens and bacteria translocation, it also lowers the absorption of nutrients [24,56], consequently, can interfere with productivity and enhance severity of clinical signs.

### 3.4. *Clostridium perfringens*

Necrotic enteritis in poultry is a frequently reported disease condition caused by the abundant growth of *Clostridium perfringens* in the intestine. *C. perfringens* strains are characterized by the production of major toxins (alpha, beta, epsilon and iota), many of these toxins have been demonstrated to contribute to the virulence of bacteria and to play a key role in the pathogenesis of animal infections [109]. Producing different toxins increases the flexibility of *C. perfringens* in causing disease under varying host conditions [110]. Recently, it was found that the majority of *C. perfringens* isolates from chickens with clinical signs of necrotic enteritis carry the necrotic enteritis B-like toxin (NetB). Earlier, it was believed that proliferation of *Clostridium perfringens* and production of alpha-toxin is the major factor for necrotic enteritis (NE) in poultry; however, NetB has recently been shown to be an essential virulence factor in clinical necrotic enteritis in broiler chickens [110,111,112,113]. NetB is a cytotoxic, haemolytic, pore-forming toxin for avian cells and it was revealed that the development of necrotic enteritis in chickens was dependent on the ability to produce NetB [111]. Beside the clinical form of *C. perfringens* necrotic enteritis in poultry, the subclinical form has also been described in the field, characterized by a damaged intestinal mucosa, decrease in digestion and absorption and reduced performance [114,115,116]. Subclinical infections are coinciding with a reduction in growth performance and negatively impacting productivity, without being recognized and treated [110].

**Figure 3:**
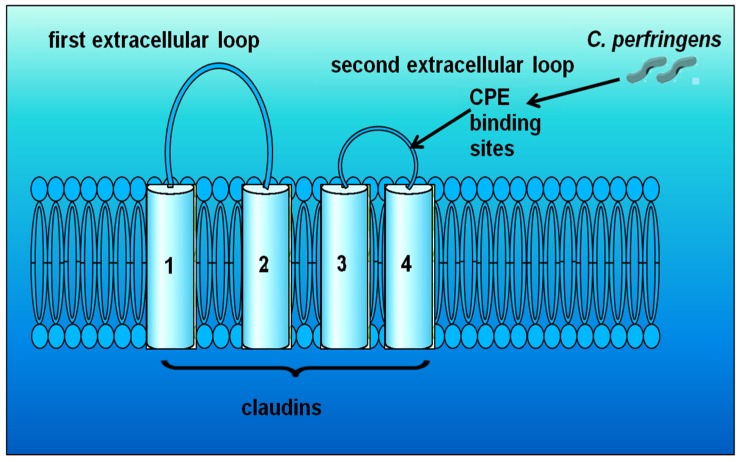
Claudins-3 and -4 are the sites of *Clostridium perfringens* enterotoxin (CPE) binding (adapted from Günzel and Yu [121]).

Many pathogens impair junctional structures indirectly by activation of signaling cascades of host cells. However, *Clostridium perfringens* enterotoxin (CPE) uses TJ proteins directly as cell surface receptors to attach [117]. CPE, a cytotoxic, pore-forming toxin, uses the claudin family as cellular receptors and it has been shown that it attaches to claudin-3 and claudin-4 of MDCK cell monolayers (Figure 3) [118,119,120,121]. Similarly, Saitoh et al. [122] revealed that CPE can bind to specific claudins, resulting in the disintegration of TJs and an increase in the paracellular permeability across epithelial cell layers. Additionally, Singh et al. [123] showed that CPE can also interact with other TJs like occludin, following binding of the enterotoxin to its receptors (claudins) in Caco-2 cell monolayers. In another study, the application of CPE to basolateral membranes was found to affect the tight junction structure by inducing a fragmentation of tight junctions within 1 h [124]. After binding, CPE damages the membrane permeability and leads to calcium influx into the cell, resulting in cell damage [125].

Nava and Vidal [126] demonstrated in human intestinal cells that an infection with a *C. perfringens* type C strain induced a significant drop on TEER and this change was mediated by redistribution of TJs protein occludin and Claudin-3. In rats, it was shown that the phospholipase C activity of the alpha toxin impaired the intestinal mucosal barrier and increased the permeability of the intestine through activation of phospholipase (Table 5) [127]. In chickens, it was reported that mucosal addition of *C. perfringens* alpha toxin can impair the intestinal mucosal barrier [128,129]. Finally, Collier et al. [130] observed that the paracellular permeability was higher in tissues from chickens infected with *C. perfringens*.

## 4. Impaired Barrier Function and Growth Performance

In poultry, the effects of enteric pathogens are not always obvious, but even in those cases where chickens do not show clinical symptoms, they may have negative effects on feed consumption, growth, immune system and other health parameters. Since it was shown that the damage of the intestinal barrier may increase the passage of pathogens to access the underlying lamina propia and activate the host’s immune compartment, impaired nutrient absorption which results in the availability of the necessary growth substrate for the proliferation of pathogens [69,70].

For instance, it was reported that the exposure to bacterial endotoxin (e.g., *Escherichia coli* or *Salmonella typhimurium*) affects the birds’ performance by a reduction in body weight and a worsening of feed conversion rate [7]. Furthermore, some studies showed that *Campylobacter* negatively impacted poultry production by a reduction of body weight with consequences on the well-being of chickens [5,95,96]. Additionally, subclinical necrotic enteritis was estimated to result in a 12% reduction in body weight and a 10.9% increase in Feed conversion ratio (FCR) compared with healthy birds, imposing a significant economic burden on the poultry industry worldwide [131]. It was estimated that losses due to altered body weight and FCR associated with subclinical necrotic enteritis range from US$878.19 to US$1480.52 per flock (20,000 birds) to reach the market weight [131].

The mechanisms by which enteric pathogens affect growth through the impaired gut barrier may comprise: (1) interference with protein synthesis and degradation; (2) alteration of the intestinal integrity (damage of intestinal villi) and disruption of the normal activity of nutrient transporters, resulting in reduced nutrient absorption; (3) increase of nutrients available for luminal pathogen proliferation, resulting in an amplification of the severity of infection; (4) increase of the maintenance requirements of the gut (for immune function needs) and thus decrease nutrient availability for the host; and (5) increase nutrient loss (decrease digestibility) by interfering with digestive enzyme synthesis and/or activities [69,70,96,132].

Finally, enteric pathogens (subclinical infections) are an important concern to the poultry industry because of production losses, reduced welfare of birds and increased risk of contamination of poultry products for human consumption, leading to high economic losses.

## 5. Restoration of the Impaired Barrier Function

Intestinal leakage, as a result of increased paracellular permeability, is a dominant feature within the pathophysiology of many enteric pathogens and could ultimately lead to an increased translocation of intestinal bacteria into the body. Therefore, impaired gut barrier function needs to be restored. Many strategies have been used for restoration either via dietary management or immunotherapy (stimulates or restores the ability of the immune defense system to counteract infection). In this context, it could be demonstrated that dietary fiber exerts beneficial effects in the gut through its bacterial metabolite, the short-chain fatty acid butyrate [133]. It has also been demonstrated that a supplementation of the chicken diet by either prebiotic, probiotic or synbiotic has an impact on barrier function [134,135,136,137,138,139,140,141,142,143,144]. It was shown that these feed additives act as quantitatively available substrates for the gastrointestinal microflora within the gut of the host [145]. They also enhance the growth of beneficial bacteria (*Bifidobacterium* and *Lactobacillus*), inhibit the growth of pathogenic bacteria like *Escherichia coli* and *Salmonella* spp., and, in consequence, improve the microbial balance in the gastrointestinal tract [146,147,148]. It was also reported that functional oligosaccharides could ameliorate the adverse effects on barrier integrity caused by heat stress in chickens [54]. In addition, the gut microbiota (commensal) itself is known to modulate barrier function which could be a potential therapeutic target [149]. This is supported by the finding that certain strains of *Lactobacilli* could reduce the permeability by increasing the relocation of occludin and ZO-1 tight junction in duodenal epithelial cells [150].

Similarly, a particular focus has been elicited on prevention of necrotic enteritis in poultry caused by *Clostridium perfringens* by the use of microbes (*Bacillus* and *Lactobacillus*) or microbe-derived products (yeasts) [151]. Liu et al. [152] found that dietary supplementation of exogenous lysozyme decreased the *C. perfringens* colonization and improved the intestinal barrier function of chickens. Furthermore, it was demonstrated that a prebiotic product (arabinogalactan Fibregum) was effective in controlling NE [153].

Linking protein synthesis with intestinal barrier permeability reflects an important feature [154,155,156,157]. Amino acids are not only important substrates for protein synthesis they are critical in supporting gut barrier integrity and function. Thereby, amino acids supplementation can be useful for alleviating intestine injuries. Thus far, attention was drawn towards glutamine offering a beneficial effect on the intestinal mucosa and gut function, which can be explained by its capability to act as an important energy source, similar to glucose [158]. It was evidenced that glutamine deprivation causes Caco-2 cell injury [159], whereas glutamine supplementation protects Caco-2 cells from barrier dysfunction [160]. Additionally, dietary supplementations with host defense peptides (HDPs) were recently shown to enhance mucosal barrier function directly by inducing the expression of TJ proteins and indirectly by displaying an improvement in nutrient utilization and a reduction in *Clostridium spp.* and coliform bacteria in the intestinal tract of broiler chickens [161].

Some immune-based therapies were designed to reduce intestinal inflammation and subsequent systemic immune activation in mice. Such immune activations were associated with reduced microbial translocation and enhanced expression of gut-junction genes. Recently, several studies have been focused on anti-inflammatory therapy which could block pro-inflammatory pathways. Some studies showed that a flavonoid (Nobiletin^®^) exerted significant anti-inflammatory effects via downregulation of inducible nitric oxide synthase (iNOS) and cyclooxygenase 2 (COX-2) expressions in vivo in rats and cell cultures (BV-2 or Caco-2) [162,163]. Therefore, basic research has to continue to provide insight into the ultimate strategies to be favored in order to restore the barrier function and to protect against onset and progression of enteric infections, coinciding with inflammation.

## 6. Importance of the Chicken Intestinal Epithelial Barrier

Sustaining a healthy gut is a prerequisite for efficient performance of farm animals, especially poultry with its high growth rate. The gut plays a key role in the digestion and absorption of nutrients and it constitutes one of the main entrance gates exposed to external factors that can challenge the bird’s health. The intestinal epithelial barrier serves as the first boundary of defense between the organism and the luminal environment. It consists of a continuous monolayer of intestinal epithelial cells which are connected together by an intercellular junctional complex limiting the space between adjacent cells. This minimizes the access of pathogens and toxins to spread into the host. Substantial evidence indicates that intestinal barrier dysfunction is considered as etiological factor in the pathogenesis of some enteric diseases [164]. Furthermore, paracellular ions and nutrient permeation is restricted by the presence of tight junctions and consequently can affect the intestinal absorptive function.

Thus, tight junction proteins play a dominant role in barrier formation. However, it is still a matter of debate how the paracellular barrier of the chicken intestine is organized, horizontally and vertically, to support a strict compartmental separation on the one hand and the transepithelial transport rates on the other hand. Thus, more knowledge on the composition of tight junction proteins in chickens are fundamental for understanding pathogenic pathways, further supporting a primary role of the epithelium tight junction in the pathogenesis of intestinal enteric pathogens and emphasizing the importance to maintain a healthy and effective intestinal barrier. In addition, chickens are an important source of zoonotic enteric pathogens. Therefore, elucidating the changes of mucosal barrier during enteric pathogens is crucial and may help in providing new tools to restore the intestinal barrier functions during infection.

## 7. Conclusions

Tight junctions are formed at the lateral sites of the cell and regulate the paracellular passage of molecules. However, not all tight junctions, consisting of multiple proteins, are merely tight as some tight junction proteins build their own transport pathways by forming channels selective for small cations, anions, or water, resulting in epithelial surfaces of different tightness. Tight junctions are regulated in their molecular composition, ultrastructure and function by intracellular scaffolding proteins and cytoskeleton. Such a cascade of interaction is not only part of cellular physiology and various adaptation processes, it can also be impaired by different microorganisms. Chronic infections with certain enteric pathogens can compromise intestinal barrier function and activate a systemic response which consequently could reduce growth efficiency.

Although the intestinal barrier and intestinal permeability are important for health and disease, the mucosal barrier and its role in enteric disease are still poorly defined in chickens. Therefore, future studies should aim to elucidate the molecular basis of the differential responses of the chicken gut to infections with certain microorganisms, which is critical for bird’s health. The improvement of food safety would be an additional surplus. Based on existing data, it can be concluded that modulation of microbiota with probiotics for repairing the gut barrier reflects a promising approach that warrants future investigations to minimize the effects of enteropathogenic microorganims in poultry.

## Figures and Tables

**Table 1:** In vitro and in vivo methods for measuring intestinal permeability.

Procedure	In Vivo	In Vitro	Reference
direct measurement of intestinal permeability	Cr51-EDTA (0.34 kDa) FITC dextran (4 kDa)	FITC dextrans (4–2000 kDa) Fluorescein (0.38 kDa) Horseraddish peroxidase (44 kDa) Mannitol (0.18 kDa) Trans-epithelial resistance	Bjarnason et al. [8] Nighot et al. [9] Awad et al. [10]
indirect measurement of intestinal permeability	TJ proteins LPS (plasma or serum) LPS binding protein	-	Bjarnason et al. [8]

**Table 2:** Interaction of enteropathogenic *Escherichia coli* with tight junctions.

Pathogen/Mechanism	In Vivo/In Vitro	Effects	Reference
EPEC dephosphorylates and dissociates occludin	in vitro	contraction of the perijunctional actomyosin ring increase in paracellular permeability and perturbing tight junction barrier function	Simonovic et al. [58]
EPEC redistributes occludin	in vivo	disruption of ion transport perturbation of intestinal barrier function	Shifflet et al. [84]
EPEC induces redistribution of ZO-1 and occludin	in vivo	increase in paracellular permeability change of tight junction structure	Zhang et al. [82]
EPEC alters the distribution of the TJ protein ZO-1	in vitro	alteration of barrier and transport functions	Philpott et al. [77]

**Table 3:** Interaction of *Campylobacter* with tight junctions.

Pathogen/Mechanism	In Vivo/In Vitro	Effects	Reference
*C. jejuni* (NCTC 12744) disrupts epithelial barrier function	in vivo	perturbation of TJ by increasing intestinal permeability	Awad et al. [5]
*C. jejuni* 81116 induces redistribution of occludin	in vitro	decrease in transepithelial electrical resistance	Dodson [90]
*C. jejuni* 81–176 induces translocation of commensal bacteria via a lipid raft-mediated transcellular process	in vivo	promotes the translocation of non-invasive bacteria across the intestinal epithelium	Kalischuk et al. [93]
*C. jejuni* RM1221 alters the distribution of the tight junction protein claudin-4	in vitro	increase in transepithelial permeability	Lamb-Rosteski et al. [91]
*C. jejuni* (NCTC 12744) interferes with intracellular Ca^2+^ signaling	in vivo	alteration of barrier and transport functions facilitates the translocation of *E. coli*	Awad et al. [69,70]

**Table 4:** Interaction of *Salmonella* with tight junctions.

Pathogen/Mechanism	In Vivo/In Vitro	Effects	Reference
*Salmonella* Enteritidis compromises the intestinal epithelium barrier	in vitro	decrease in the trans-epithelial ion conductance	Awad et al. [20]
*Salmonella* Typhimurium decreases in claudin-1, claudin-4, and occludin mRNA proteins expression	in vivo	disruption of the epithelial barrier function	Shao et al. [105]
*Salmonella* Typhimurium decreases claudin-1 and occludin mRNA expression	in vivo	alteration of the intestinal mucosal barrier function	Zhang et al. [100]
*Salmonella* Typhimurium decreases the mRNA expression of both ZO-1 and occludin, causes a redistribution of both epithelial TJ proteins claudin-1 and ZO-2	in vitro	damage of the intestinal barrier function facilitates the translocation of pathogenic and non-pathogenic bacteria	Koehler et al. [103]

**Table 5:** Interaction of *Clostridium perfringens* with tight junctions.

Pathogen/Mechanism	In Vivo/In Vitro	Effects	Reference
*C. perfringens* type C causes a redistribution of epithelial TJ proteins occludin and claudin-3	in vitro	decreases the trans-epithelial electrical resistance	Nava and Vidal [126]
*C. perfringens* alters epithelial TJs barrier through activation of phospholipase	in vivo	perturbation of TJ by an increased intestinal permeability	Otamiri [127]
*C. perfringens* decreases claudin-1 and occludin mRNA expression	in vivo	alteration of the intestinal barrier function by increasing intestinal permeability	Collier et al. [130]
*C. perfringens* enterotoxin targets directly TJ protein claudins as receptors	in vitro	impairment of TJ barrier function increase in paracellular permeability	Saitoh et al. [122]
